# Gonorrhœal Iritis

**Published:** 1901-04-20

**Authors:** 


### GONORRHŒAL IRITIS.

We are so accustomed to associate iritis with syphilis that it is as well to draw attention to a paper read by Dr. John Griffith before the Harveian Society, in which he maintained that gonorrhœa was a common cause of iritis, and in fact a more common cause than syphilis. Not only was it a complication—it was also a sequel of gonorrhœa. Cases occurring at a remote period were, he thought, overlooked as regards their causation, and being often recurrent were regarded as rheumatic; the fact being that both the recurrent iritis and the rheumatic symptoms were due to attacks of urethritis. It was his experience that cases of recurrent iritis in syphilitic subjects had been associated with rheumatism and a history of urethritis. The importance of this in regard to treatment, and especially in regard to the avoidance of unnecessary treatment, is sufficiently obvious.

